# Disruption of cellular homeostasis induces organelle stress and triggers apoptosis like cell-death pathways in malaria parasite

**DOI:** 10.1038/cddis.2015.142

**Published:** 2015-07-02

**Authors:** S Rathore, G Datta, I Kaur, P Malhotra, A Mohmmed

**Affiliations:** 1 Department of Biotechnology, All India Institute of Medical Sciences, New Delhi, India; 2 International Centre for Genetic Engineering and Biotechnology, New Delhi, India

## Abstract

A regulated protein turnover machinery in the cell is essential for effective cellular homeostasis; any interference with this system induces cellular stress and alters the normal functioning of proteins important for cell survival. In this study, we show that persistent cellular stress and organelle dysfunction because of disruption of cellular homeostasis in human malaria parasite *Plasmodium falciparum*, leads to apoptosis-like cell death. Quantitative global proteomic analysis of the stressed parasites before onset of cell death, showed upregulation of a number of proteins involved in cellular homeostasis; protein network analyses identified upregulated metabolic pathways that may be associated with stress tolerance and pro-survival mechanism. However, persistent stress on parasites cause structural abnormalities in endoplasmic reticulum and mitochondria, subsequently a cascade of reactions are initiated in parasites including rise in cytosolic calcium levels, loss of mitochondrial membrane potential and activation of VAD-FMK-binding proteases. We further show that activation of VAD-FMK-binding proteases in the parasites leads to degradation of phylogenetically conserved protein, TSN (Tudor staphylococcal nuclease), a known target of metacaspases, as well as degradation of other components of spliceosomal complex. Loss of spliceosomal machinery impairs the mRNA splicing, leading to accumulation of unprocessed RNAs in the parasite and thus dysregulate vital cellular functions, which in turn leads to execution of apoptosis-like cell death. Our results establish one of the possible mechanisms of instigation of cell death by organelle stress in *Plasmodium*.

Malaria is a major healthcare problem worldwide resulting in an estimated 0.65 million deaths every year. Present strategy of malaria control is totally dependent on pharmacological treatments and there is a constant need to identify new drug targets involved in important metabolic pathways in the parasite.^1^ The cellular machinery responsible for protein quality control and folding is essential for cellular homeostasis and survival of eukaryotic cells. The protein quality control is particularly important for malaria parasites because of its high replication rate, high temperature stress and high load on endoplasmic reticulum (ER) because of large amount of proteins that are to be secreted or exported to the host cytosol. In eukaryotic cells, inhibition of 26 S proteasome is one of the major causes for low clearance of unfolded proteins from ER and therefore leads to ER stress. ER stress response may help the cell to survive through the stress, it can also trigger apoptosis when high levels of unfolded proteins persist for a longer time.^2^ We have earlier shown that disruption of an important metabolic pathway of the parasite can incite the parasite to undergo apoptosis-like cell death.^3^ A number of other studies have suggested that apoptosis-like cell death can be induced in *Plasmodium falciparum* by different anti-malarial drugs, antibiotics and other small molecules.^4, 5^ However, the mode of induction of cell death and different cascade of molecular/cellular events leading to apoptosis-like cell death in the parasite are not clearly understood.

In this study, we have assessed cellular stress induced by proteasome inhibition on asexual stage *P. falciparum* parasites. Global quantitative proteomic analyses identified putative pro-survival pathways in the parasites under cellular stress. We further show that persistent proteasome inhibition cause parasite cell death, which is mediated by a cascade of molecular and cellular events. Overall, our results highlight a probable mechanism of cell death and survival in *Plasmodium* under cellular stress.

## Results

### Proteasome inhibition in *P. falciparum* leads to apoptotic-like cell death

**Figure 1:**
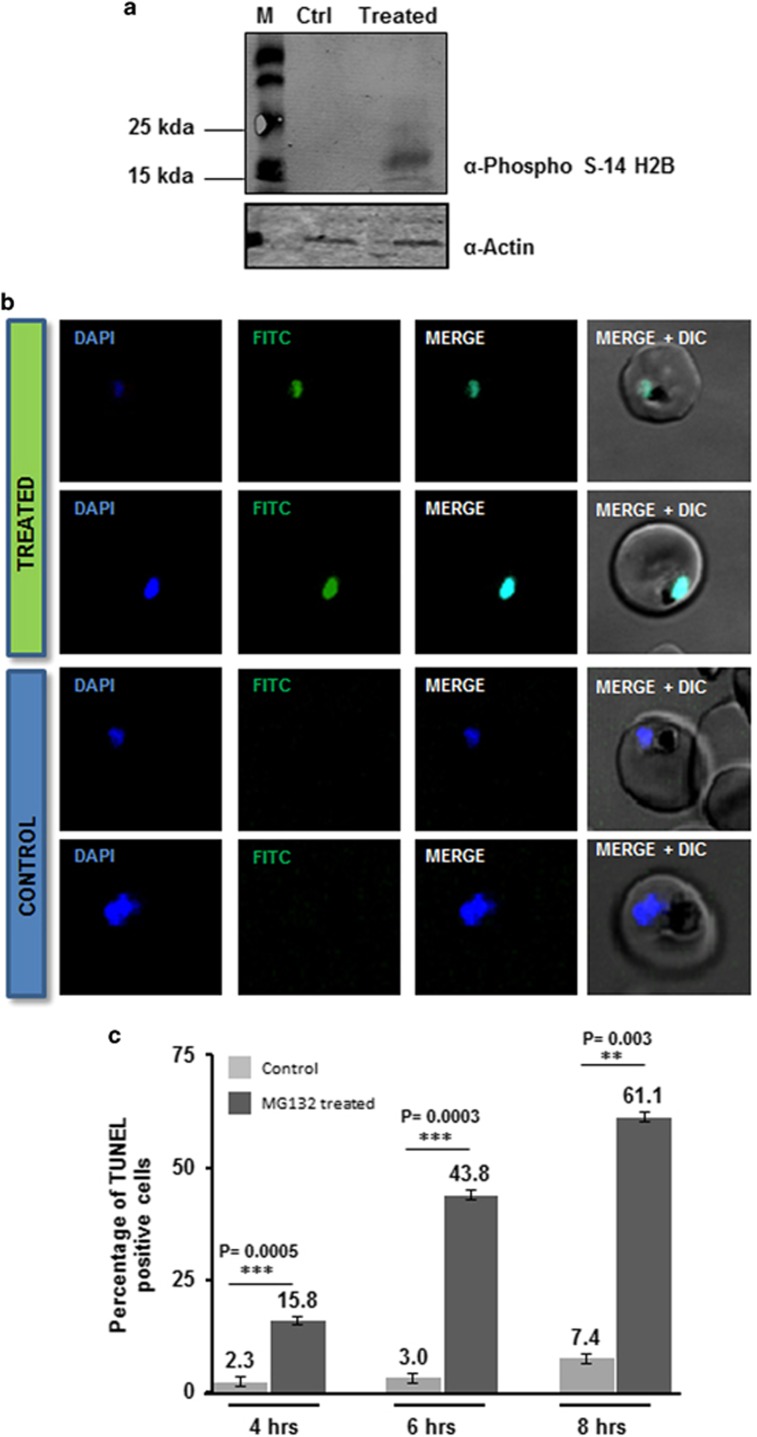
Markers of apoptosis-like cell death in parasites treated with MG132 (MG132). (**a**) Western blot analysis showing phosphorylation (anti-phospho S14) of histone H2B in MG132-treated parasites (lane 2) as compared with control (lane 1), actin antibodies were used as loading control. (**b**) DNA fragmentation in *P. falciparum* parasites as assessed by TUNEL staining, fluorescent microscopic images of TUNEL-positive parasites (TdT staining) after treatment with MG132 as compared with control. (**c**) Bar graph showing percentage of TUNEL-positive parasites (*n*>100) at different time points (4, 6 and 8 h) after treatment with MG132. **P<0.01 and ***P<0.001

We assessed the effect of the 26 S proteasome inhibitor, MG132, on asexual blood stage *P. falciparum*. MG132 can inhibit parasite growth in a dose-dependent manner (EC_50_ ~50 nM) (Supplementary Figure S1A). In the treated cultures, majority of parasites showed developmental abnormalities, delayed growth and were not able to grow beyond healthy trophozoites (Supplementary Figure S1C); subsequently, the parasite cytosol became completely condensed and parasites appeared as densely stained structures (Supplementary Figure S1C). We observed about 55% of parasites showed this ‘crisis form morphology' within 10–15 h after treatment (Supplementary Figure S1B). These parasites remained as pyknotic forms in the culture and were not able to develop into schizonts and progress further (Supplementary Figure S1B). Removal of the inhibitor at 1–4 h after treatment resulted in almost complete reversal of growth inhibition (Supplementary Figure S1D). However, a very small percentage parasite population was able to recover when the inhibitor was removed at 6 or 8 h after treatment (Supplementary Figure S1D). The reversibility of growth inhibition, and the characteristic morphological abnormalities in the treated parasites suggested apoptotic-like cell death. To investigate this, we analyzed for cellular markers of the programmed cell death (PCD) such as histone 2B phosphorylation^6^ and nuclear DNA fragmentation. Phosphorylation at serine 14 (phosS14) is known to be an epigenetic marker of apoptotic cells.^7^ The western blot analyses using anti-phosSer14 H2B antibody showed increased amount of phosS14 in the treated parasites (Figure 1a). Analysis of nuclear DNA fragmentation using terminal deoxynucleotidyl transferase (TdT) dUTP nick end labeling (TUNEL) staining was found to be positive for parasites in the treated cultures only after 6 h of treatment (Figures 1b and c).

### Cellular homeostasis proteins get upregulated before onset of cell death

**Figure 2:**
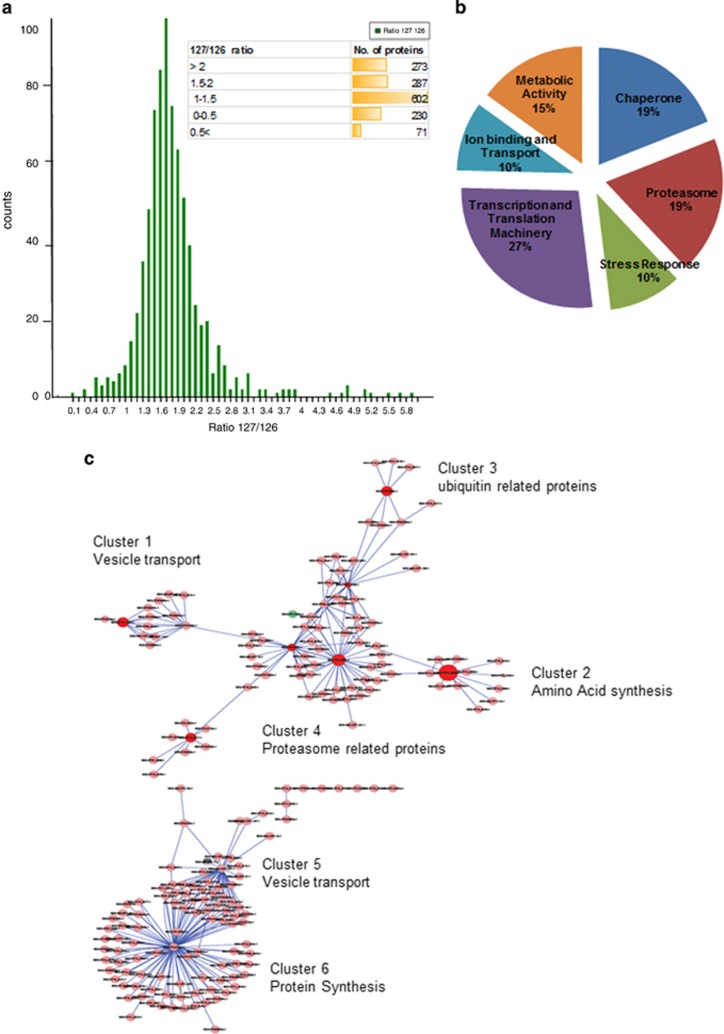
Quantitative proteomic analysis reveals increase in levels of cellular homeostasis proteins at 4 h of proteosomal inhibition. (**a**) Histogram and table (inset) showing the distribution of number of proteins having different peptide ratios (127/126; MG132 treated/control) as estimated by isobaric tagging based MS quantification. (**b**) Pie chart showing the percentage of upregulated proteins belonging to different functional classes based upon selected gene ontology (GO) terms from KEGG pathway. (**c**) Functional association network of selected clusters of proteins that are upregulated after MG132 exposure. The network shows probable linkage between the vesicle-transport, signaling and metabolic clusters

We analyzed global proteomic profile in these treated parasites before point of no return (4 h). Of the soluble protein samples, 1463 proteins were confidently identified and 861 proteins were found to be differentially expressed in response to treatment (Figure 2a). About 65% of these differentially expressed proteins were found to be upregulated (e.g. 127/126 ratio >1.5) (Figure 2a). A total of 270 proteins were found to be related to proteasome function (19% of the identified proteome) and 142 proteins related to cellular stress (10% of the identified proteome). The other major group of proteins identified in the analysis were related to transcription and translational machinery (Figure 2b); in addition, a number of chaperone proteins (~19% of the identified proteome) were upregulated in the treated parasites. A substantial proportion of the upregulated proteins are hypothetical proteins.

We observed increased levels of ER proteins such as Sec61 alpha and eIF2 alpha (Supplementary Figure S7B). Bax-inhibitor (BI-1), which has a 'pivotal role' as a pro-survival factor during ER stress in mammalian cells, was also found to be upregulated (Supplementary Figure S6B). We also found a number of upregulated proteins known to be involved in vesicular trafficking and endosomal pathways, these include SNAREs, Rab5a, Rab7 and Vps16 (Supplementary Figure S6B). The interacting protein–protein network for all upregulated proteins were downloaded from STRING database. Using Cytoscape and Mcode Pluggin, we looked for different clusters associated with the key proteins. This interaction network showed that upregulated target proteins were majorly associated with vesicular trafficking and proteasome (Figure 2c, Supplementary Figures S5, S6A and S7A).

### Proteasome inhibition leads to organelle stress

**Figure 3:**
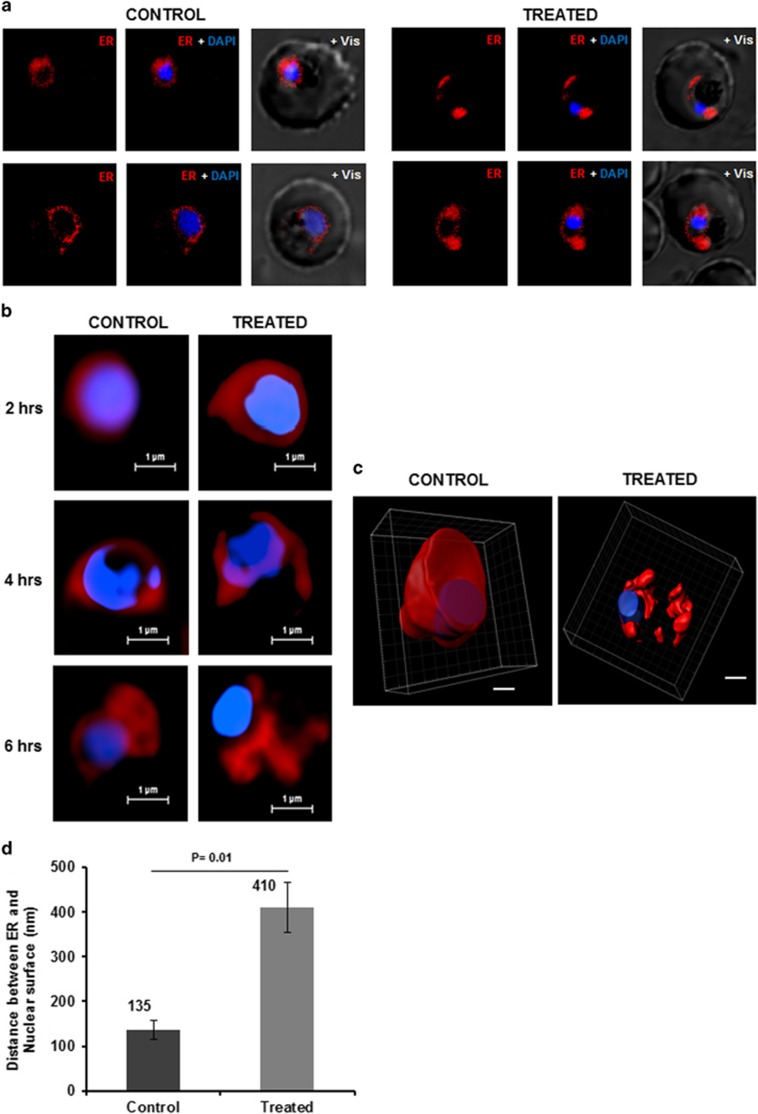
Altered morphology of ER and release of stored calcium in *P. falciparum* parasites under prolonged proteosomal inhibition. (**a**) Fluorescent microscopic images of *P. falciparum* parasites stained with ER Tracker Red at 6 h after treatment with MG132. The parasite nuclei were stained with DAPI (blue). (**b**) Super resolution microscopic images at different time points (2, 4, 6 h) after MG132 treatment. The parasite showed a disrupted ER network at 4 and 6 h as compared with control that showed a continuous ER around the nucleus. (**c**) 3D images reconstructed using Z-stacks of ER Tracker stained control and treated parasites. (**d**) Bar graph showing increase in distance between nuclear and ER surface as assessed by super-resolution microscopic images in MG132-treated parasites as compared with control

**Figure 4:**
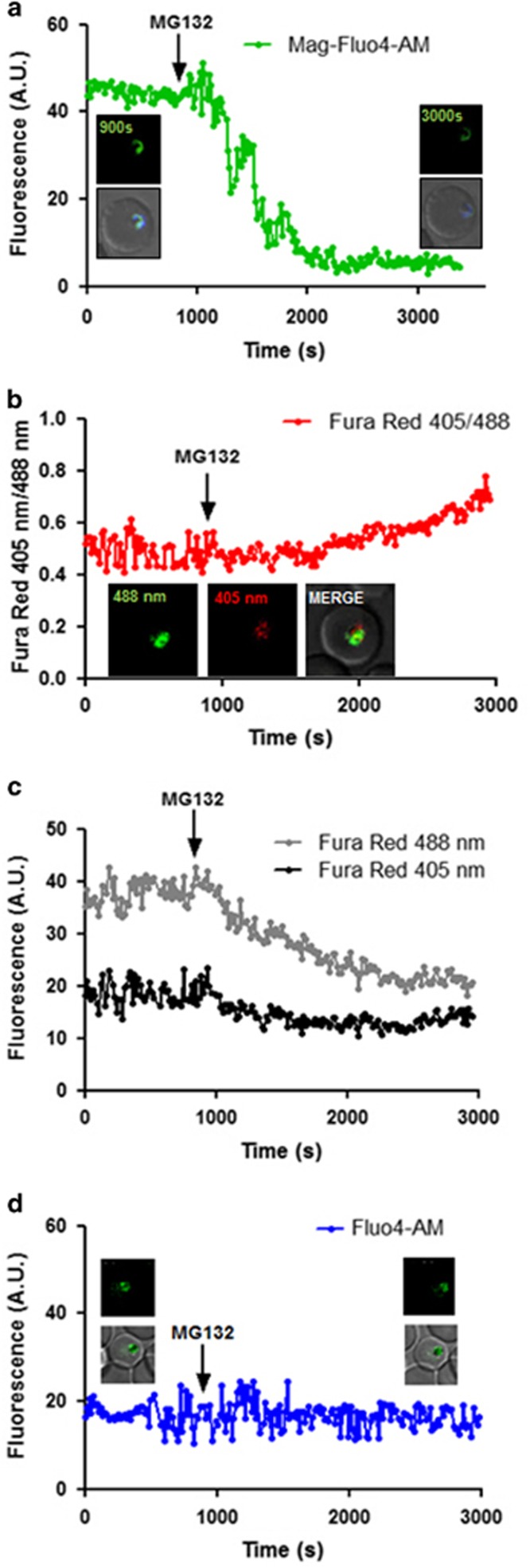
ER stress-associated Ca^2+^ kinetics by confocal fluorescence microscopy. (**a**) Kinetics of free calcium release from the ER upon MG132 treatment as assessed by Mag-Fluo-4 AM staining. Parasites at early trophozoite stage were loaded with Mag-Fluo-4 AM and fluorescence intensity was plotted as a function of time. Treatment with MG132 (100 nM) resulted in a rapid loss of ER-free calcium. The experiments was repeated for 20 iRBCs in two independent experiments; a single selected representative graph and image (inset showing colocalization with ER Tracker Blue white) is shown. (**b**) Confocal live cell imaging with Fura-Red AM staining shows that cytoplasmic-free Ca levels rise after MG132 treatment (100 nM). A representative parasite's graph and image is presented here. The fluorescence ratio (405/488 nm) increases following MG132 addition. (**c**) Graph showing decrease in Fura-Red fluorescence at 405 and 488 nm for the above parasite. (**d**) Fluo-4 AM staining to demonstrate that there is no decrease in digestive vacuole calcium concentration during this period of MG132 treatment. The fluorescence intensity before and after addition of MG132 remains nearly constant. Additional fluorescence graphs and images are included in Supplementary Figure 2

Our data showed that persistent proteasome inhibition beyond reversible time point induces apoptosis-like cell death in *P. falciparum*. In order to understand involvement of cellular organelles in parasite cell death after MG132 treatment, we studied the morphological and developmental effect on different parasite organelles at different time points after the treatment. Confocal microscopic studies showed morphological abnormalities in shape and structure of ER within 2 h after the treatment (Figure 3a). Detailed analysis of these parasites by super-resolution microscopy and 3D reconstruction of the confocal images showed that the ER is extensively enlarged and becomes a loose mesh-like network in the treated parasites, which is apparently detached from the nucleus (Figures 3b and c). We also estimated extent of this expansion/detachment by measuring the distance of ER from nucleus, which was found to be much higher in the treated parasites as compared with control (Figure 3d). The release of Ca^2+^ from ER stores, an event potentially triggered by ER stress, critically affects the survival of various cells by inducing pro-apoptotic stimuli. We measured the Ca^2+^ transients in the ER by using the low-affinity Ca^2+^ indicator Mag-Fluo-4 AM (*K*_d_ 22 *μ*M); for the food vacuole (FV) and cytoplasm we used the high-affinity Ca^2+^ indicators Fluo-4 AM (*K*_d_ 345 nM) and Fura-Red AM (*K*_d_ 140 nM), respectively. The fluorescence values for Mag-Fluo-4-AM in live cell imaging showed that the Ca^2+^ concentration in ER rapidly decreased after MG132 treatment (Figure 4a, Supplementary Figure S2A). A decrease in Fura-Red AM fluorescence at both excitation wavelengths was observed upon MG132 addition, whereas the ratio F405 nm/488 nm showed increase during the same time interval suggesting increase in Ca^2+^ concentration in the cytoplasm (Figures 4b and c, Supplementary Figure S2C). However, no change in Fluo-4 AM fluorescence inside the FV was observed (Figure 4d, Supplementary Figure S2B). These results show that MG132 treatment leads to a loss in ER calcium.

**Figure 5:**
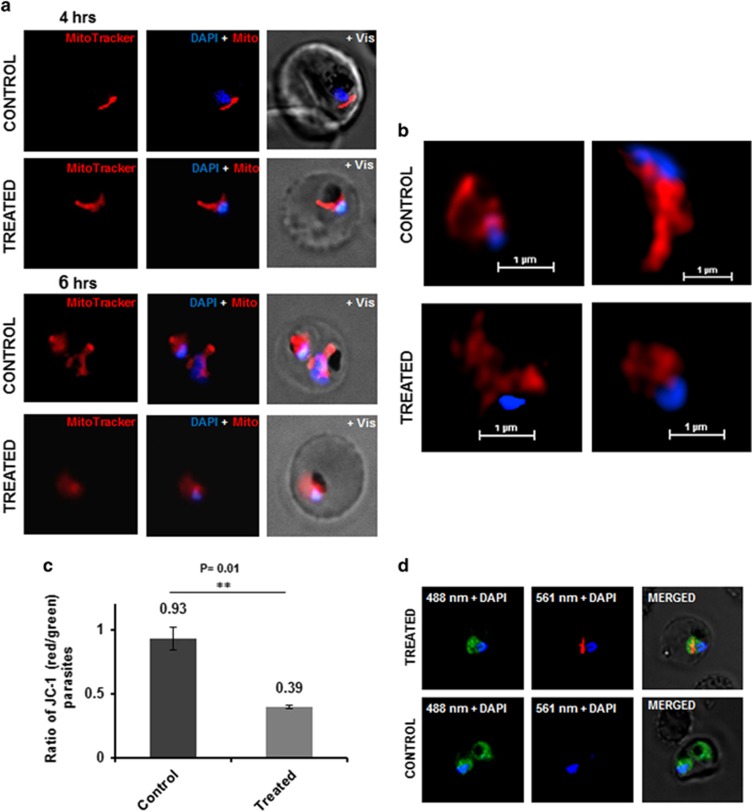
Developmental abnormalities in mitochondria and loss of Δ*ψ*_m_ in *P. falciparum* parasites after MG132 treatment. (**a**) Fluorescent microscopic images of parasites treated with MG132 or solvent alone (control) and stained with MitoTracker Red dye at different time points (4 h and 6 h) after treatment. The parasite nuclei were stained with DAPI (blue). The control parasites show elongated and branched mitochondria, whereas MG132-treated parasite showed deformed mitochondria after 6 h of treatment. (**b**) Super resolution microscopic images of MitoTracker stained parasites showing deformed mitochondria after 6 h of MG132 treatment as compared with control parasites. (**c**) Bar graph showing reduction in ratio of JC-1 (red)/JC-1 (green) in parasite population after treatment with MG132; a total of one million cells were counted by flow cytometry to calculate JC-1 ratio. (**d**) Fluorescent microscopic images of JC-1-stained representative parasites showing accumulation of aggregated JC-1 (red) in the mitochondria and monomeric JC-1 (green) in the cytosol. The parasite nuclei were stained with DAPI (blue). **P<0.01

Microscopic studies with parasite labeled for apicoplast (D10 ACP-GFP) showed that there was no significant morphological change in these organelles with 2–4 h of treatment as compared with control parasites (Supplementary Figure S4). However, at 4 h of treatment the mitochondria in the treated parasites showed diffuse mitotracker staining and disintegrated structure as compared with control parasites, which had intact branched mitochondria (Figures 5a and b). In addition, MG132 treatment caused a significant decline in mitochondrial membrane potential (Δψ_m_) as compared with control (Figures 5c and d). Overall, these studies show that two cell death-associated organelles develop morphological abnormalities before the cell death is initiated in the treated parasites.

### Organelle stress in parasite activates caspase-like cysteine protease

**Figure 6:**
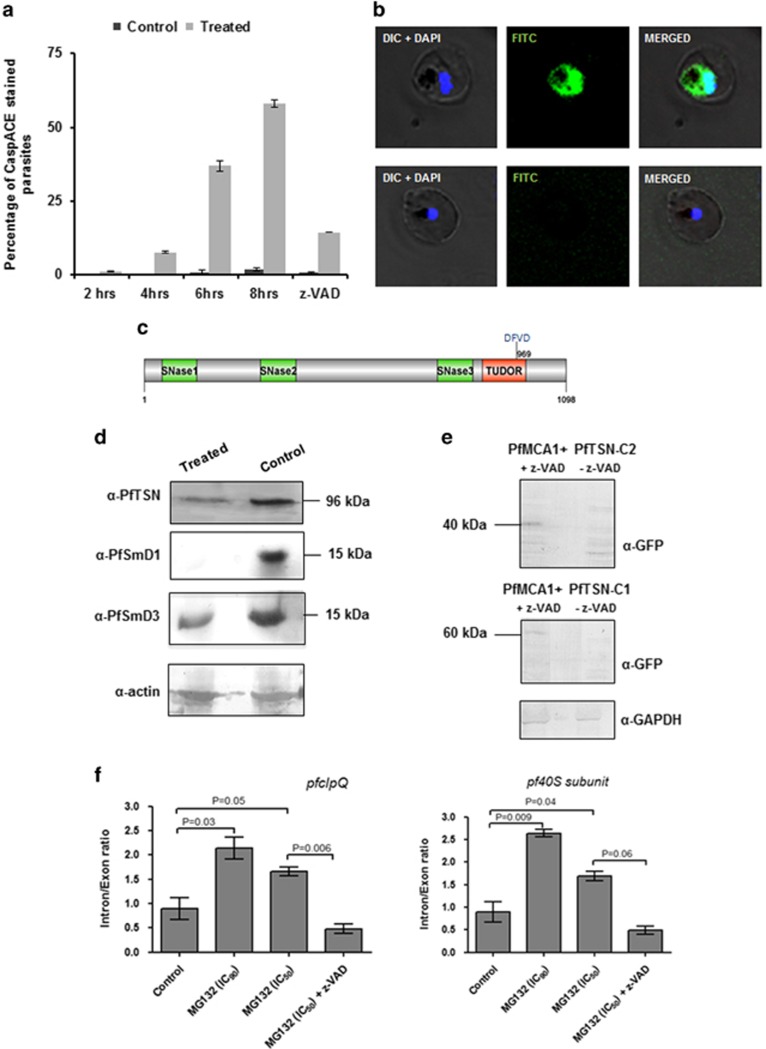
Organelle stress leads to activation of VAD-FMK (CaspACE) binding cysteine proteases, which in turn downregulates RNA-splicing machinery. (**a**) Graph showing percentage of CaspACE tagged parasites in the cultures at different time points (2, 4, 6 and 8 h) after treatment with MG132. A large number of parasites were CaspACE-positive after proteasome inhibition (6 h and 8 h after treatment). (**b**) Fluorescent microscopic images showing CaspACE labeling (green) in the treated parasites as compared with control. (**c**) Schematic diagram of PfTSN protein showing domain architecture and location of putative metacaspase cleavage site. (**d**) Western blot analyses showing levels of PfTSN, PfSmD1 and PfSmD3 proteins in the treated parasites (lane 1) as compared with control (lane 2). (**e**) Western blot analyses of HUH-7 cells showing degradation of PfTSN-C1 and PfTSN-C2 when co-expressed with PfMCA1, which is inhibited by z-VAD-FMK treatment. (**f**) Graphs showing intron/exon ratio of two different genes as assessed by quantitative RT-PCR analysis in MG132-treated cultures (EC_50_ and EC_90_) as compared with controls. Increase in intron/exon ratio in the treated parasite suggests reduction in mRNA processing; however, pre-treatment with VAD-fmk inhibitor reversed this effect

Our results with parasite morphology, development and proteomic studies showed that the proteasomal inhibition causes ER stress-like phenotype in the parasites. We further examined downstream pathways that led to apoptosis-like cell death in the treated parasites. The MG132-treated parasites (50 nM ~EC_50_) showed activation of a small population of CaspACE-positive cells at 4 h of treatment; after 4 h the percentage of parasites showing CaspACE labeling increased significantly reaching ~35% at 6 h and ~60% at 8 h after treatment (Figures 6a and bSupplementary Figure S3A). However, at time point earlier than 4 h there was no CaspACE-stained parasite population in the treated cultures as compared with the control, suggesting that activation of VAD-FMK-binding proteases occur only >4 h after the treatment.

### Activation of VAD-FMK-binding cysteine proteases leads to downregulation of RNA-splicing machinery

Caspases are known to be the major proteins in the pathway of PCD by acting on various important proteins needed for cell survival, which include several components of transcription and splicing machineries.^8^ *P. falciparum* harbors caspase-related cysteine proteases, metacaspases.^9^ Recently, Tudor Staphylococcus Nuclease (TSN) has been identified as one of the substrates for these activated metacaspases.^8^ We assessed TSN protein levels in these parasites with activated VAD-FMK-binding caspase-like cysteine proteases. Levels of PfTSN protein are drastically reduced in MG132-treated parasites after activation of VAD-FMK-binding proteases as compared with control parasites. In addition, we also assessed levels of two other important components of splicing machinery in *Plasmodium,* that is, PfSmD1 and PfSmD3 also found to be reduced in these parasites cells (Figure 6d). Overall, components of the splicing machinery are downregulated in stressed parasites probably due to cleavage by activated cysteine proteases.

To demonstrate direct interaction between PfTSN and PfMCA1, we expressed two fragments of PfTSN (PfTSN-C1 and PfTSN-C2) along with full-length PfMCA1 in Huh-7 hepatoblastoma cell lines (Supplementary Figure S3B). We co-expressed PfMCA1 with PfTSN-C1 fragment and PfMCA1 with PfTSN-C2 in Huh-7 cell line and analyzed by western blotting. We observed a number of bands in lanes corresponding to degraded fragments of PfTSN-C1 and PfTSN-C2. The intact PfTSN-C1 and PfTSN-C2 (i.e. 60kDa and 40 kDa bands) were observed when the CaspACE inhibitor z-VAD-FMK was added (10 *μ*M); this shows inhibition of PfMCA1 activity by z-VAD-FMK (Figure 6e).

### Unprocessed mRNA levels accumulates in stressed parasites

We further assessed changes in the levels of unprocessed and processed mRNA in the treated parasites. Quantitative RT-PCR analyses was carried out to estimate intron/exon ratio for a given gene. The *pfclpQ* RNA in the treated parasite (<EC_50_ ~50 nM) showed a intron/exon ratio nearly double as compared with the control parasites (Figure 6f); similarly, RNA of 40 S ribosomal subunit also showed a similar effect having intron/exon ratio nearly twice as compared with control (Figure 6f). These effect on RNA processing in stressed parasites can be reverted when the parasites were treated simultaneous with caspase inhibitor (z-VAD-FMK); in these parasite the intron/exon ratio was restored to nearly normal (Figure 6f), clearly indicating the role of caspase-like proteases in downregulation of RNA-splicing machinery. However, the effect on RNA processing in stressed parasites were not ubiquitous; there was no change in intro/exon ratio for two other genes that we analyzed, *pfhrp2* and *pfhsp90* (data not shown).

### Activation and reversal of VAD-FMK-binding cysteine proteases associates with parasite cell death

**Figure 7:**
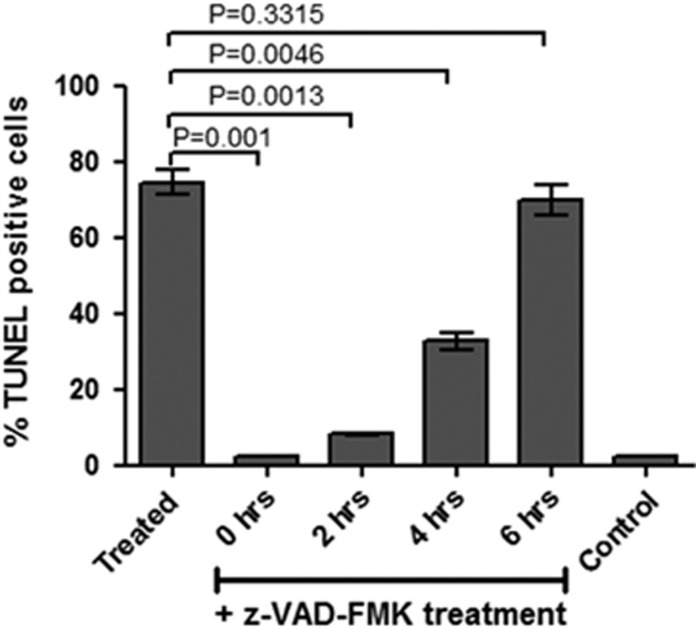
Association between activation of VAD-FMK-binding proteases and apoptosis-like cell death in *P. falciparum*. Bar graph showing percentage of TUNEL-positive parasites in cultures treated with z-VAD-fmk at different time points (0, 2, 4 and 6 h) after MG132 treatment as compared with cultures treated with MG132 alone

Pre-incubation of treated parasites with z-VAD-FMK was able to revert the percentage CaspACE-positive population in the treated parasite to normal levels as in the control set (Figure 6a); further, z-VAD-fmk was able to reduce the splicing inhibition levels in MG132-treated parasites (Figure 6f). Therefore, z-VAD-FMK was used to treat parasites at different time points after proteasome inhibition and reversal of cell death was assessed using TUNEL assay. The MG132-treated parasites were able to overcome the cell death by Z-VAD-FMK treatment, which support role of caspase-like VAD-FMK-binding proteases in the parasite cell death after organelle stress because of proteasomal inhibition. However, when caspase inhibitor was added at 6 h after treatment, a large percentage of parasite population was not able to recover from cell death (Figure 7).

## Discussion

Maintenance of cellular homeostasis and integrity of cellular proteome depends upon protein turnover including stability, refolding and degradation of the proteins. During asexual blood stage cycles of malaria parasites, there is high load on protein synthesis and turnover because of rapid multiplication rate of the parasite; therefore, proper functioning of protein quality control machinery is essential for maintenance of cellular homeostasis. In this study, we analyzed molecular and cellular events in the *P. falciparum* parasite after induction of organelle stress by proteosomal inhibition, MG132. Developmental arrest at the trophozoite stage and the appearance of pyknotic forms in MG132-treated parasite cultures, points toward apoptosis-like cell death as described earlier.^3^ *Plasmodium* cell death remains a topic of discussion since recent past; a number of studies have tried to understand the mode of apoptosis-like cell death and its machinery in the parasite.^10, 11^ One of the main characteristic of apoptosis-like cell death is a programmed cascade of committed events with a point of no return.^11, 12^ Our data from wash-off experiments showed that proteasomal inhibition induced parasite death can be reversed by removal of the inhibitor within 4 h of treatment; however, after that time point the cells are committed to die, this may suggest that it is also programmed event. In eukaryotic cells, the hallmarks of apoptosis-like cell death include the fragmentation of nuclear DNA and histone H2B phosS14.^13, 14^ Our results with TUNEL assay and histone modifications markers analyses indicate an apoptotic-like cell death in MG132-treated parasites.

In eukaryotic cells, the interplay between these survival and cell death responses ultimately determines the fate of the stressed cell.^15^ We first tried to assess the molecular events in parasite at early stages of stress because of proteasomal inhibition. Our results of global quantitative proteomic analysis suggest that *P. falciparum* tries to increase number of stress-related proteins after proteasome inhibition. Our data showed increase in levels of various chaperones, such as Der-1, cpn60 and Hsp70, along with various translation machinery proteins, which points toward *Plasmodium*'s efforts to combat stress. The other major group of proteins, which were found to be increased in levels were proteins of Rab family, that is, Rab1a, Rab2, Rab7 and Rab18, which are known to be associated with different vesicular trafficking pathways in *Plasmodium*.^16, 17^ Recently it was shown that in stressed *P. falciparum* parasites, the Rab7 associates with ATG8 coated vesicles.^18^ ATG8 is an important protein for formation of autophagy vesicles, auto-phagosomes, and thus ATG8 is an important component of autophagy. Autophagy in malaria parasites remains to be a topic of debate and it is not clear if true autophagy occurs in *Plasmodium*.^19, 20^ Rab7 is known to be involved in trafficking of endosomal material to lysosomal system in eukaryotic cells^21^ and toward the FV in *P. falciparum*.^18^ We have also observed increased levels of other components of autophagy machinery, ATG18, in the treated samples as compared with controls. Increase in levels of these proteins in our data points toward increased vesicular trafficking by parasite to combat stress as shown in case of other organisms.^22^

Another important protein involved in UPR signaling, that is, eIF2α was found to be upregulated after treatment with MG132. eIF2α is known to be linked between UPR signaling and stimulation of autophagy-like events; under stress the eIF2α gets phosphorylated after activation of PERK pathway. Although a clear UPR signaling pathway is not present in *P. falciparum*, a putative homolog of PERK is present in the parasite.^23, 24^ Another set of interesting proteins, which are found to be upregulated in the treated parasites include ER-associated Sec61 and Bax-inhibitor. Earlier studies have provided evidence that the Sec61 channel mediates passive calcium efflux from the intact ER^6, 25^ and also during ER stress.^26^ Signal peptide peptidase (SPP) was found to be quntitatively reduced in the treated parasite. Eukaryotic SPPs are multi-pass integral membrane proteins from the aspartyl protease family that cleave transmembrane substrates.^27, 28^ PfSPP inhibition has been shown to reduce the parasites ability to cope up with ER stress, and could have a significant role in deciding the survival of parasite.^29^ Taken together, our results showing increased eIF2a α along with increased vesicular trafficking components, may also be one of the like survival strategy in the parasite under stress.

It has been reported that proteasome inhibition may induce autophagy via UPR-dependent pathways.^30^ However, the machinery required for UPR pathways is absent in *Plasmodium*.^24^ Upregulation of autophagy-related proteins in MG132-treated parasites may suggest that autophagy-like mechanism may be a survival mechanism in *Plasmodium* under stress in a UPR-independent manner. A recent study also suggests that the parasite directs vesicles/organelle fragments to the FV in starved parasites.^18^ Overall, quantitative proteomics studies, at 4 h, the time point at which parasite retain the ability to revert back to normal growth, suggest that the parasite experiencing stress are able to device a survival strategy. These strategies may help the parasites to survive initial stress and revive if the stress is removed; however, if the stress continues further the cell undergoes apoptosis-like PCD.

As our results pointed out the relation between organeller stress and cell death in the parasite, therefore in this study we also tried to analyze effect of cellular stress on various organelle. Recent study from Chaubey *et al.* have shown that induction of ER stress in *Plasmodium* parasite by exposure to a reducing agent for short period, leads to gametocytogenesis in the next growth cycle. However, in our study, we showed if the ER stress persists beyond point of no return, parasite can initiate cell death processes. The ER is also an important site for calcium storage within the cell, which is coupled with its quality control machinery to produce correctly folded protein.^31^ We found the induction of ER stress simultaneously caused release of stored Ca^2+^ in the parasite cytosol. Subsequently, another important organelle, mitochondria in the parasite, also get stressed, which loses its membrane potential and ability to divide further. Eventually, we observed activation of caspase-like VAD-FMK-binding proteases in the stressed parasite. The *P. falciparum* genome does not contain any classical caspase-like protein, however, caspase-like activity is also described during apoptosis in other organisms that lack classical caspases.^32, 33, 34, 35^ *P. falciparum* genome harbors three metacaspase-like proteases that are closely related to eukaryotic caspases, these proteases contains C14 domain with a catalytic dyad of cysteine an histidine as in case of caspases.^36^ Metacaspases are known to have essential role in during PCD in plants;^37, 38^ the TSN is shown to be a substrate of activated metacaspases in plants.^8^ The TSN is a conserved proteins that is known to be involved in regulation different transcription and translation steps.^39^ The TSN homolog in *P. falciparum*, PfTSN, is shown to be an important functional component of spliceosome Sm core complex.^40^ PfTSN harbors the DFVD motif near its C-terminus, which is a probable cleavage site for caspase-like enzymes. We observed reduction of PfTSN levels in parasites after activation of caspase-like z-VAD-FMK-binding proteases; along with PfTSN, other associated nuclear proteins like PfSmD1 and PfSmD3, which are part of nuclear splicing machinery, were also found to be reduced. Indeed, most of the TSN partner proteins in transcription and splicing are also known targets to be cleaved by caspases in mammalian cells. Degradation of PfTSN and other associated nuclear proteins suggested downregulation of normal RNA processing machinery in these stressed parasites. Our quantitative PCR analysis confirmed accumulation of unprocessed RNAs in these parasites. Overall, our data suggest that downregulation of splicing and hence translation of proteins may dysregulate vital cellular machinery in the parasite, ultimately leading to cell death.

To ascertain the role of caspase-like VAD-FMK-binding proteases in these steps, we inhibited activity of these proteases in MG132-treated parasites. Pre-incubation of treated parasites with z-VAD-FMK was able to revert the percentage CaspaACE-positive population in the treated parasites and reduced the splicing inhibition levels. Further, treatment with z-VAD-FMK before activation of VAD-FMK-binding proteins also reverted the cell death and TUNEL staining; however, the cells were not able to recover from cell death after complete activation of caspase-like VAD-FMK-binding proteases, which suggest that these activated protease already initiated the cascade of reactions that led to apoptosis-like cell death. This set of data show association of parasite recovery by removal of proteosomal inhibitor at different time points and activation of caspase-like VAD-FMK-binding proteases.

Overall, our data points toward one of probable PCD mechanisms induced because of proteasomal inhibition in *P. falciparum*, which occurs through ER stress. Disruption of normal protein turnover and cellular homeostasis in the parasite lead to dysregulated protein response at the ER, which acts through activated caspase-like proteases, and inhibition of the splicing machinery in the parasite ultimately causing apoptosis-like cell death.

## Materials and Methods

### Parasite culture and growth inhibition assay

*P. falciparum* strain 3D7 was cultured with 4% hematocrit in RPMI media (Invitrogen Corp., San Diego, CA, USA) supplemented with 10% albumax-hypoxanthine using a protocol described previously.^41^ To assess the effect of MG132 on *P. falciparum* growth, ring stage parasite culture was synchronized by sorbitol treatment. Synchronous culture at ring stage (6–8 h.p.i.) at 2% parasitemia was incubated with MG132 (1* μ*M, 100 nM, 10 nM and 1 nM concentrations in triplicate) in 24-well flat-bottomed cell culture plate (NUNC) along with artemisnin at 20 nM concentration (in triplicate) as positive control. A control well was also set up with solvent only, that is, DMSO as negative control. Thin smears were made from each well at different time points and stained with Giemsa for microscopic analysis. After 48 h of incubation, the numbers of ring stage parasites per 5000 RBCs were determined and percentage ring stage parasitemia was calculated to assess the parasite growth. Parasite growth was also assessed by DNA fluorescent dye-binding assay (SYBR green) following Smilkstein *et al.*^42^ Graph was plotted and the effecter concentration for half maximum response was estimated (EC_50_) was calculated using GraphPad Prism software (La Jolla, CA, USA). To study the reversal of effects of MG132, treated parasites were washed twice with incomplete media after the indicated time points and placed back into complete media without inhibitor. For further assays, dissecting the mechanism of ER stress, early-stage trophozoites (24–28 h) were incubated with MG132 at EC_50_ for given time points.

### Mammalian expression plasmid constructs, transfection and western blotting

To generate HUH cell expression plasmid constructs, two fragments of PfTSN one corresponding to middle fraction of gene (C1, 603 aa-1099 aa), and other representing the C-terminal half (C2, 870 aa–1099 aa) were amplified from the cDNA DNA using primers 1124A (5′-AAGCTTATGGAATATATTAATTTAAACGAA-3′) and 1126A (5′-CCGCGGATTTTCATCATCATAGTCGAT-3′) for middle fragment, and 1127A (5′-AAGCTTATGAATGATTTAACAGCTTATGATAAT-3′) and 1128A (5′-CCGCGGATTTTCTGTAGGTATTTTTAAACCTGC-3′ for C-terminal fragment. The amplified PCR products were digested with *Hind*III and *Sac*II restriction enzymes and cloned into with *Hind*III and *Sac*II sites of peGFP-N1 vector. The resultant plasmids were labeled as peGFP-N1 – PfTSN M and peGFP-N1 – PfTSN C, respectively. Similarly, PfMCA1, full gene (1 aa–600 aa) was amplified from genomic DNA using primers (forward 5′-AGATCTTATAAAGCTTATGGAAAAAAATACGTC-3′ reverse 5′-AAGCTTTATAGAATTCAAAAAAAAATAAATTTTTAGG-3′). The amplified PCR products were digested with *Bgl*II and *Hind*III restriction enzymes and cloned into with *Bgl*II and *Hind*III sites of pCMV-6A vector. The resultant plasmids were labeled as pCMV-6A – PfMCA1. The HUH cells were cultured in Dulbecco's modified Eagle's medium (Invitrogen Corp.) with 10% heat-inactivated fetal calf serum in a humidified CO_2_ (5%) incubator at 37 °C. Fresh monolayers of 40 to 60% confluent HUH cells growing in 35-mm diameter wells were transfected with 2 to 4 *μ*g plasmid DNA using Jet Prime Plus reagent (Invitrogen Corp.) following manufacturer's instructions. To detect cleavage of PfTSN by PfMCA1, cells were harvested 48 h post transfection and the lysate was analyzed using anti-GFP and anti-c-myc mouse monoclonal antibodies (Roche Inc., New York, NY, USA). Anti-GAPDH antibodies were used as in loading control experiments. The PfTSN fragments expression was confirmed by western blotting using anti-GFP antibodies (60 kDa for PfTSN-C1 and ~40 kDa for PfTSN-C2; Figure 6e). PfMCA1 expression was similarly confirmed using anti-FLAG antibodies. We observed two bands at ~66 kDa and ~58 kDa representing the unprocessed and processed forms of PfMCA1, respectively (Supplementary Figure S3B) as reported earlier.^43^

### Isolation of total RNA, cDNA preparation and quantitative RT-PCR

Synchronized parasite cultures at of early ring stage parasites (6–8 h.p.i.) was treated with 100 nM MG132 and parasites were harvested after 30 h. After Saponin lysis, equal volume TRIzol reagent (Invitrogen Corp.) was added and total RNA was purified as described by the manufacturer, the total RNA was treated with DNase I. An aliquot of 50 ng of total RNA was used to synthesize cDNA using cDNA synthesis kit (Invitrogen Corp.) following manufacturer's recommendations. Set of gene-specific primers specific for their intron and exon regions were designed using Beacon Designer 4.0 software (Palo Alto, CA, USA), for the genes *pfhrp2* (intron: 907 A: 5′-GATAACGTAAGCATTTTAATTGC-3′, 908 A: 5′-GTTATTATTAAATGCGGAATTATTC-3′ exon: 909 A: 5′-CCGCATTTAATAATAACTTG-3′, 910 A: 5′-ATGTGCTTGAGTTTCGTG-3′), *pfclpQ* (intron: 911 A: 5′-GATTGATGAATATCCAAG-3′, 912A: 5′-GTTACAAAAAAGGAAAGGTAC-3′ exon: 913 A: 5′-AAGTTGTGTTGAGTTAGC-3′, 914 A: 5′-TAAAACATCACCATTACC-3′), *pf40S* ribosomal subunit (intron: 915 A: 5′-TACGTGGGTCAAAAAATGG-3′, 916 A: 5′-TAACACAAAGACAATGCG-3′ exon: 917 A: 5′-AACTCTGCTTACCGTAAATG-3′, 918 A: 5′-CACTTCTACCAAATCCTGAG-3′), PFC0270w (intron: 919 A: 5′-GAATATGAAATGAGGATATAC-3′, 920 A: 5′-AAAGATTTGTAACTATCGAGTG-3′ exon: 921 A: 5′-GAAATTACAAATAAATGGGC-3′, 922 A: 5′-TGAAACTTGTAGATTATCG-3′) and 18 S rRNA control primers (18SF: 5′-GCTGACTACGTCCCTGCCC-3′ 18SR: 5′-ACAATTCATCATATCTTTCAATCGGTA-3′) were used following Blair *et al.*^44^ Quantitative real-time PCR was carried out in triplicate using the iCycler version 3.0 (Bio-Rad, Hercules, CA, USA); each reaction was containing equal amount of cDNA, 100 ng of both sets of the gene-specific primers and 1 × SYBR Green PCR mix (Bio-Rad). PCR reactions were performed as follows: 56 °C primer annealing and 65 °C template extension for 35 cycles on a Lightcycler 6500 (Bio-Rad). Threshold cycle (Ct) values were calculated by using iCycler software (Bio-Rad). Standard curves for each gene were obtained by using different dilutions of wild-type gDNA (100 to 1 ng) as template, and these standard curves were used to determine genome equivalents of Ct values for respective gene and 18 S rRNA in each RNA sample. Genome equivalents of each gene were normalized using that of 18 S rRNA for all the RNA samples. The *P*-values were calculated by Student's *t*-test.

### Isobaric mass tag labeling of trypsin-digested parasite proteins and peptide fractionation

Parasites treated with solvent alone or MG132 for 4 and 8 h were harvested using 0.15% saponin in the presence of protease inhibitor cocktail (Roche Inc.) and lysed in equal volume of urea buffer (urea-8M, Tris-Cl pH 8.2 50 mM and NaCl-75 mM) and incubated on ice for 30 min. The supernatant was separated from the debris by centrifugation and the total protein was estimated using Pierce BCA Protein Assay Kit (Thermo Fisher Scientific Inc., Waltham, MA, USA) as per the supplier's protocol. Equal amounts of proteins from both sets were reduced using 5 mM DTT (Sigma, St. Louis, MO, USA) at 56 °C for 25 min to reduce disulfide bonds. Protein mixtures were cooled to room temperature (RT) and then alkylated with 14 mM iodoacetamide (Sigma) for 30 min in dark at RT. Additional iodoacetamide were quenched with 5 mM DTT for 15 min at room temperature in dark. Proteins were diluted to the final urea concentration of 1M with Tris-Cl, pH 8.2 and were digested with trypsin (Promega, Mannheim, Germany, cat. no. V5111 or V5113) at 37 °C overnight. Digestion reactions were stopped by addition of tri-fluoro acetic acid (Sigma). Tryptic-digested peptides were desalted using reverse-phase C18 SepPak solid-phase extraction cartridges from Waters (Bangalore, India) for removal of salt and urea.

These desalted peptides were used for Isobaric Mass Tag labeling using TMT Isobaric Mass Tagging Kits (Thermo Scientific, Waltham, MA, USA) as per manufacturer's protocol. Briefly, desalted peptides from control and MG132-treated parasites were reconstituted in 100 mM TEAB (triethyl ammonium bicarbonate) and incubated with TMT labels 126 and 127, respectively, for 1 h at RT and the reaction was stopped by addition of 8% hydroxylamine. The labeled peptides from both sets were mixed and fractionated using HILIC chromatography (hydrophilic interaction liquid chromatography). Fifteen fractions were collected, concentrated using speed vac and analyzed on LC/MS/MS.

### Liquid chromatography tandem mass spectrometry

Tandem mass spectrometry experiments were performed using the Nano LC-1000 HPLC nanoflow system (Thermo Fisher Scientific Inc.) via nano-electrospray ion source connected to hybrid Orbitrap Velos Pro mass spectrometer (Thermo Fisher Scientific Inc.) with Acclaim PepMap100 C18 column (3 *μ*m, 75 *μ*m × 2 cm), which further connected online to Acclaim PepMap100 C18 column (2 *μ*m, 50 *μ*m × 15 cm). Peptides from each fraction were separated by a 120 min gradient of 5% buffer B to 90% buffer B (buffer B contains 0.1% formic acid in 95% acetonitrile; buffer A: 0.1% formic acid in 5% acetonitrile) with a flow-rate of 300 nl/min. Peptides eluting from the column tip were electro-sprayed directly into the mass spectrometer with a spray voltage of 1.4 kV. Data acquisition was performed in a data-dependent mode to automatically switch between MS, MS2. Full-scan MS spectra of peptides (m/z 350–1800) were acquired in the Fourier transform ion cyclotron resonance cell with a resolution of 60 000. The 20 most abundant ions were sequentially isolated and fragmented in the high-energy collisional dissociation cell. A dynamic exclusion of ions previously sequenced within 90 s was applied. All unassigned charge states and singly charged ions were excluded from sequencing. A minimum of 1000 counts was required for MS2 selection.

### Peptide identification and validation by Proteome Discoverer Software

The acquired data in the form of raw spectrum files were imported to Proteome Discoverer 1.4 (Thermo Fisher Scientific Inc.). Proteins were identified by searching against the *P. falciparum* proteome databases using SEQUEST algorithm and peptide matches were validated using Percolator (Proteome Discoverer 1.4). Search parameters for protein identification specified a mass tolerance of 20 p.p.m. for the precursor peptide (MS^1^) and 0.1 Da for fragmentation spectra (MS^2^) and a trypsin specificity allowing up to two missed cleaved sites. Carboxy-amido-methylation of cysteines was specified as a fixed modification and oxidation of methionine, deamidation of glutamine and asparagine were set as variable modifications. The TMT-duplex labeling at N-terminal, as well as on lysine residue of the peptides, was set as variable modification. The peptides and proteins were quantified using ratios of 127/126.

### Protein interaction network analysis

Proteomics data were analyzed based on Gene ontology described on *Plasmodium* genome database PlasmoDB. Further protein–protein interaction networks were derived from String protein–protein interaction network database, which is based on four different parameters from *Plasmodium*: physical interactions (yeast two-hybrid data) and indirect analysis (genomic context and co-expression data) to create the interaction network of the targeted proteins, we used Cytoscape version 2.8.1. List of Uniprot identifiers of each target protein were provided as input to the PISCQUISC web client plugin to build the network. Wherever there were multiple isoforms of the target proteins, all were taken to build the interaction network. Cytoscape and the Mcode plugin were used to identify clusters of the regulatory networks associated with differentially expressed genes.

### Isolation of parasites and western immunoblotting

Total parasite lysate were separated by SDS-PAGE under reducing conditions before proteins were transferred to PVDF membrane using a Transblot Wet transfer system (Bio-Rad) according to manufacturer's instructions. The membranes were blocked in blocking buffer (1 × PBS, 0.1% Tween-20, 5% milk powder) for 2 h. The blots were washed and incubated for 1 h with a primary antibody (rabbit anti-*Pf*TSN; rat anti-*Pf*SmD1; and rat anti-*Pf*SmD3 (in-house made)) each used at 1 : 1000 dilutions; anti-phospho S14 H2B, rabbit polyclonal (Santa Cruz Biotechnology, Dallas, TX, USA), respectively, and mice monoclonal anti-H2B (Santa Cruz Biotechnology). The secondary anti-mouse-HRP conjugate or anti-rat-HRP conjugate (Promega) antibodies were used at 1 : 3000 dilutions for the respective blots. The protein bands reacting with the antibodies were detected using the Amersham ECL detection kit (Piscataway, NJ, USA) and visualized by exposing blots to autoradiography films (Kodak, Rochester, NY, USA).

### Organelle staining and fluorescence microscopy

To visualize ER morphology, ER Tracker Red (Life Technologies, Grand Island, NY, USA) was added at a final concentration of 200 nM directly to parasite suspensions in complete culture medium and incubated with shaking at 37 °C for 20 min after which 0.1 ng/*μ*l, 6-diamidino-2-phenylindole (DAPI, Sigma) was added for further 10 min. Following three washes with 1 × PBS (pH 7.4), samples were mounted on glass slides and observed either on a Nikon A1 Confocal Microscope (Nikon Corporation, Tokyo, Japan) or Nikon A1 microscope with N-SIM (Structured Illumination Microscopy, for super-resolution). To visualize mitochondria, the parasites were stained with MitoTracker Red CMXRos (Invitrogen Corp.) as described earlier (Rathore *et al.*^45^) and the parasite was visualized using confocal fluorescence microscope.

For confocal microscopy, images were acquired with Plan Apochromat 100 × 1.40 NA oil immersion objective lens (Nikon Corporation) in NIS Elements and Z-stacks were taken for 21 steps at 200 nm intervals. For 3D-SIM images, Ti-sapphire solid state lasers (405, 488, 561 nm) provided wide field illumination and multi-channel images were captured simultaneously using three cameras Andor Technology iXon DU897 EMCCD (Andor Technology, Belfast, UK). Data were captured using a CFI Apo TIRF 100x oil (NA 1.49) lens (Nikon Corporation) and sectioned using a 200 nm Z-step size. Raw 3-phase images were reconstructed as described elsewhere. All images were captured and analyzed using NIS Elements C/NIS Elements AR. Individual Z-stacks were exported as 8-bit RGB TIFF formats and selected confocal Z-stacks were further reconstructed in Imaris (Bitplane, Zurich, Switzerland) as 3D models.

### Measurement of intracellular calcium by confocal microscopy and live cell imaging

Confocal fluorescence microscopy and live cell imaging was performed using a Nikon A1 microscope as mentioned above. To detect ER stress-associated Ca^2+^ dysregulation, we used a three Ca^2+^ fluorophores to stain different subcellular compartments. The ER Ca^2+^ was measured using Mag-Fluo-4 AM. Parasites were stained with Mag-Fluo-4 AM (10 *μ*M) and ER Tracker Blue White DPX (2 *μ*M) for 30 min at 37 C, followed by controlled saponin treatment (0.01% for 2 min) to trap the dye within ER. This sample was then transferred to a glass bottomed Petri dish kept in a stage top incubator (Tokai Hit, GM-8000, Tokai Hit Corporation, Gendoji-cho, Japan). Physiological conditions were maintained by a continuous supply of mixed gas (5% CO_2_, 5% O_2_ and balanced nitrogen). ER Ca^2+^ transients using Mag-Fluo-4 AM was then monitored in these parasites by exciting the sample at 488 nm and detecting the emission signals at 561 nm and sequential ER Tracker Blue White DPX signals were detected by excitation at 364 nm and emission at 640 nm. To detect changes in cytoplasmic Ca levels, cells were labeled by the ratiometric dye Fura-Red AM.^46^ Fura-Red is a ratiometric dye with dual excitation (488 nm for free Ca^2+^ and 405 nm for bound Ca^2+^) and single emission (640 nm). For Fura-Red, a decrease in fluorescence corresponds to an increase in Ca^2+^ concentration for excitation at both wavelengths, but the ratio F405 nm/488 nm rises with an increase in Ca^2+^ concentration.^47, 48, 49^ Parasites were loaded with Fura-Red and confocal images were acquired every 15 s by alternate excitation with 405 and 488 nm lasers. Fura-Red was alternately excited with the 405 and 488 nm lasers, and fluorescence signals were separated from excitation wavelengths using a quad-band dichroic mirror and emission filter set (405/488/561/640, Semrock Inc., Rochester, NY, USA). Images were acquired using an Andor iXon DU897 EMCCD camera every 15 s keeping the pinhole at 1.0 *μ*m. This resulted in a pair of images and the cytoplasm was marked by region of interest (ROI), which was tracked for changes in mean fluorescence intensity over time. Changes in digestive vacuole-free calcium was detected by staining with Fluo-4 AM (10 *μ*M) and imaged using the 488 nm laser and marking the DV area as ROI. All image analysis in these ROIs were carried out in the NIS Elements AR Analysis software (ver 4.13.04, Nikon Corporation) and fluorescence values were exported to MS Excel and plot using GraphPad Prism (ver 5.00).

### Measurement of caspase-like cysteine protease activation

To assess the activation of caspase-like cysteine proteases, cells were stained with CaspACE FITC-VAD-FMK *in Situ* Marker (Promega) as per manufacturer's instructions. Briefly, parasites were collected from experimental and control sets at different time points and incubated with 10 *μ*M CaspACE FITC-VAD-FMK (fluorescein isothiocyanate-valyl-alanyl-aspartyl-[O-methyl]-fluoromethylketone) in complete media for 30 min at 37 °C followed by washing with 1 × PBS. The stained samples were analyzed by flow cytometry using CellQuestPro software on FACS Calibur (Becton Dickinson, San Jose, CA, USA) to assess fluorescence staining (Em-525 nm/Ex-488 nm) of infected RBCs.

### Mitochondria membrane potential assay

The Δ*Ψ*_m_ was assessed in the parasite using MitoProbe JC-1 Assay Kit for Flow Cytometry (Molecular Probe, Eugene, OR, USA) as described earlier this kit uses a cationic dye, JC-1 (5,50,6,60-tetrachloro-1,10,3,30-tetraethylbenzimidazolylcarbocyanine iodide), which remains in monomeric form in the cytoplasm and has a green fluorescence (525 nm). However, the membrane potential of functional mitochondria establishes a negative charge that allows the lipophilic dye to accumulate and form aggregates in the mitochondria, which have red fluorescence (590 nm). Infected RBCs were collected from parasite cultures in control and experimental sets and incubated with JC-1 dye (at a final concentration. of 10 mM) for 30 min at 37 °C. Cells were washed with PBS and analyzed by flow cytometry using FACS Calibur flow cytometer and CellQuestPro software (Becton Dickinson). The infected RBCs were analyzed using green (488 nm) and red (635 nm) filters. Ratio of JC-1 (red)/JC-1 (green) were calculated to assess the loss of Δ*Ψ*_m_. The JC-1-stained uninfected RBCs were used as background controls.

### TdT-mediated dUTP nick end labeling

Parasites at early trophozoite stages were treated with either MG132 or solvent alone and incubated for different time points. The DNA fragmentation in the treated and untreated samples was assessed by TUNEL using *In Situ* Cell Death Detection Kit, TMR Red (Roche Applied Science, Mannheim, Germany), as per manufacturer's instructions. Briefly, samples were fixed with paraformaldehyde and glutaraldehyde, washed with PBS and permeabilized by treating with 0.01% Triton-X 100. Subsequently, RBCs were incubated with a mix of TdT enzyme and TMR Red labeled dUTP for 1 h at 37 °C and washed thrice with 1 × PBS. The labeled parasites were observed using Nikon A1 Confocal Microscope and the percentage of TUNEL-positive cells were calculated.

## Abbreviations

PCD: programmed cell death
MG132: Z-Leu-Leu-Leu-al
FITC-VAD-FMK: fluorescein isothiocyanate-valyl-alanyl-aspartyl-[O-methyl]-fluoromethylketone
Δ*Ψ*_m_: mitochondrial membrane potential
TUNEL: terminal deoxynucleotidyl transferase dUTP nick end labeling
