# Supplementary figures and images for: Disruption of cellular homeostasis induces organelle stress and triggers apoptosis like cell-death pathways in malaria parasite
Source: Cell Death Dis. 2015 Jul 2;6(7):e1803–. doi: 10.1038/cddis.2015.142 (PMC4650714; doi:10.1038/cddis.2015.142)

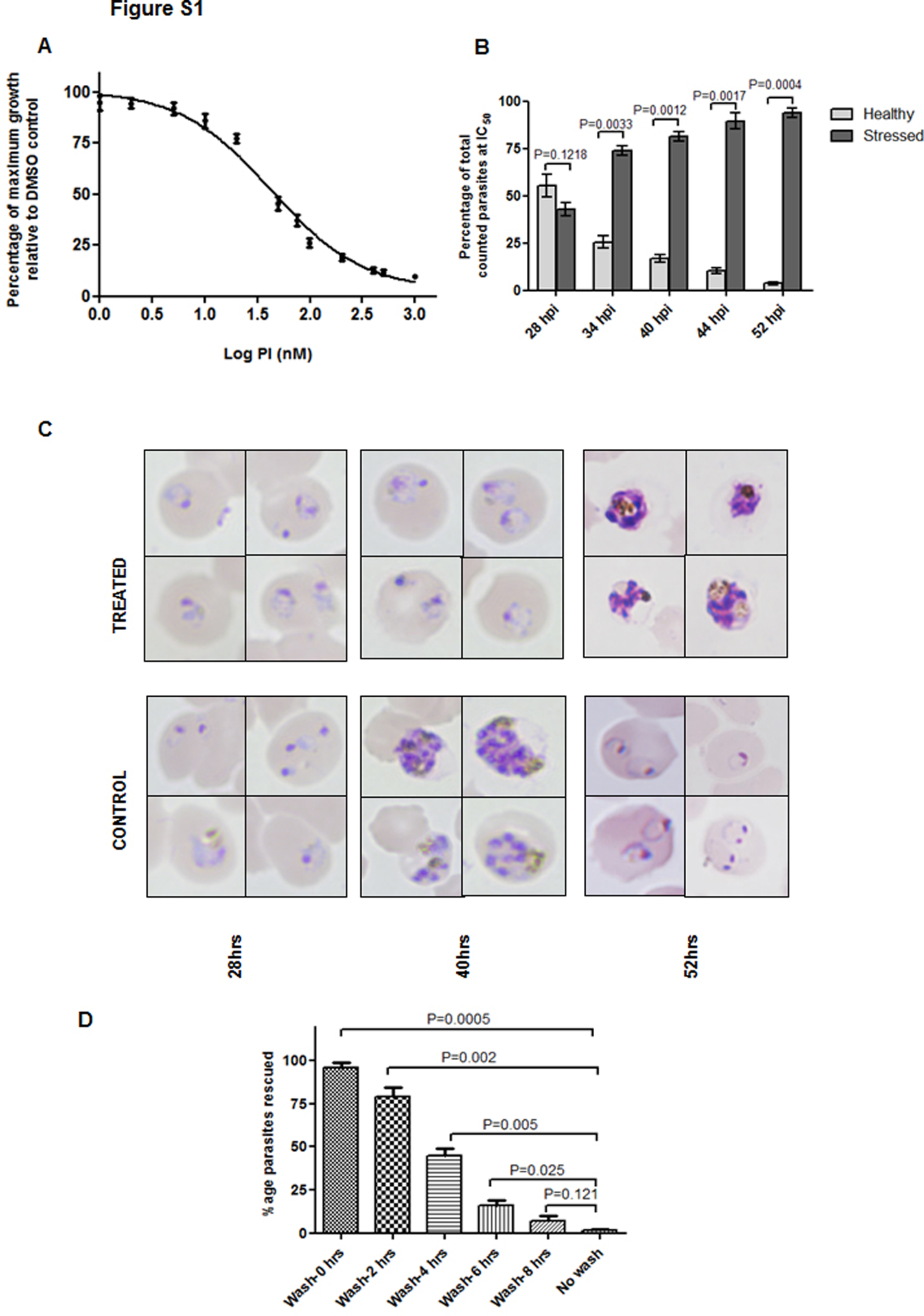

Supplement: Supplementary Figure 1 [file cddis2015142x1.tif]

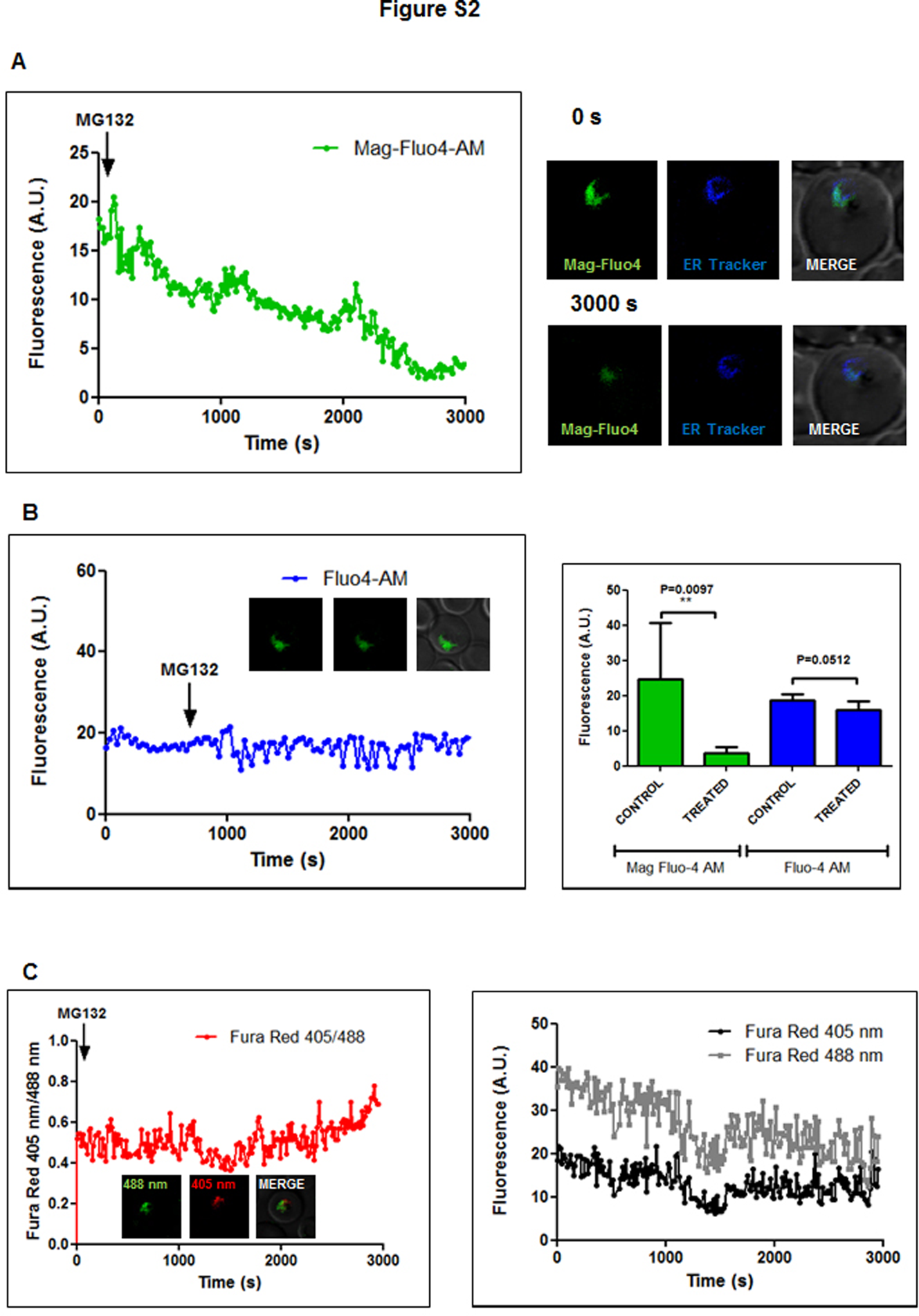

Supplement: Supplementary Figure 2 [file cddis2015142x2.tif]

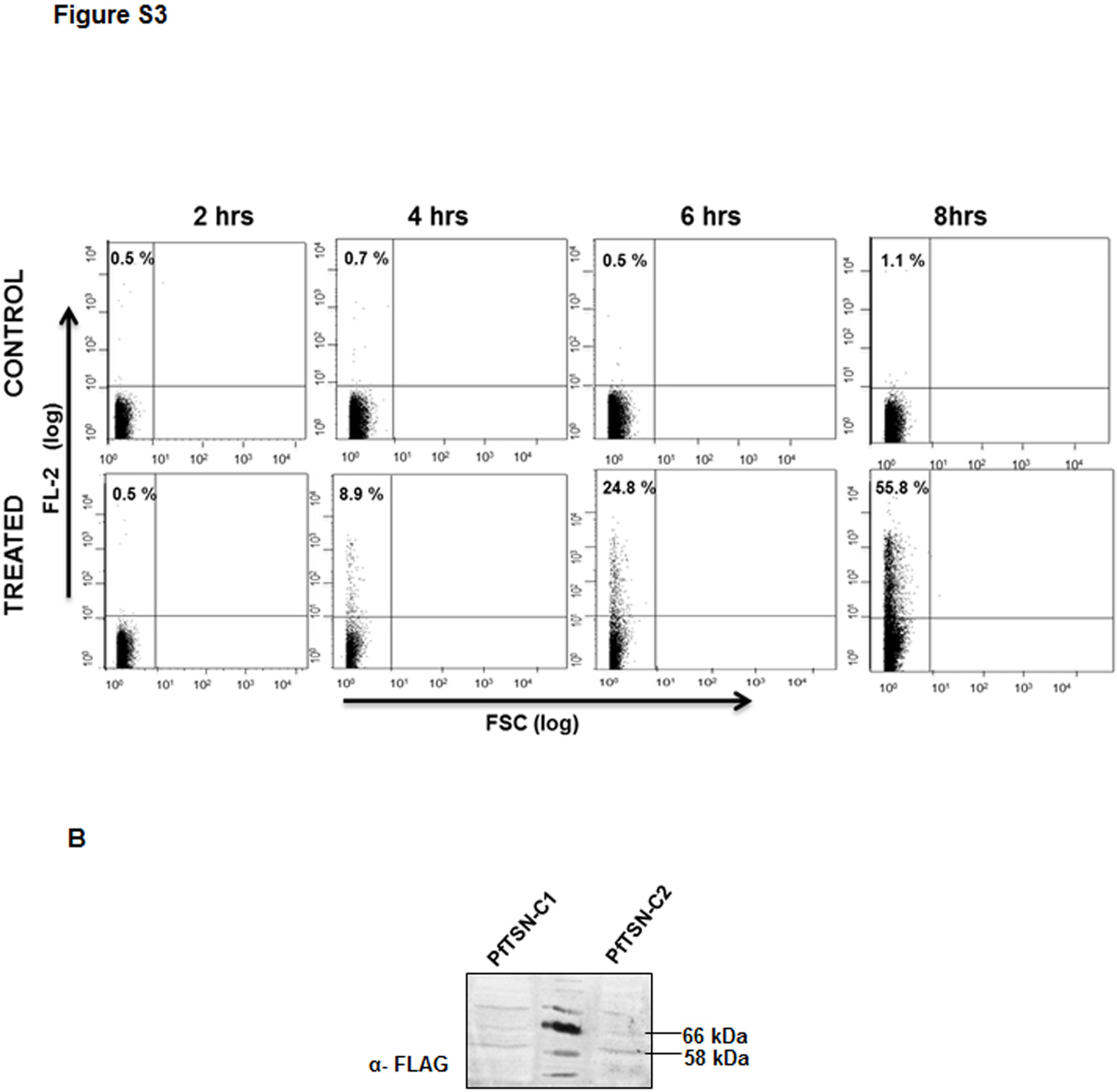

Supplement: Supplementary Figure 3 [file cddis2015142x3.tif]

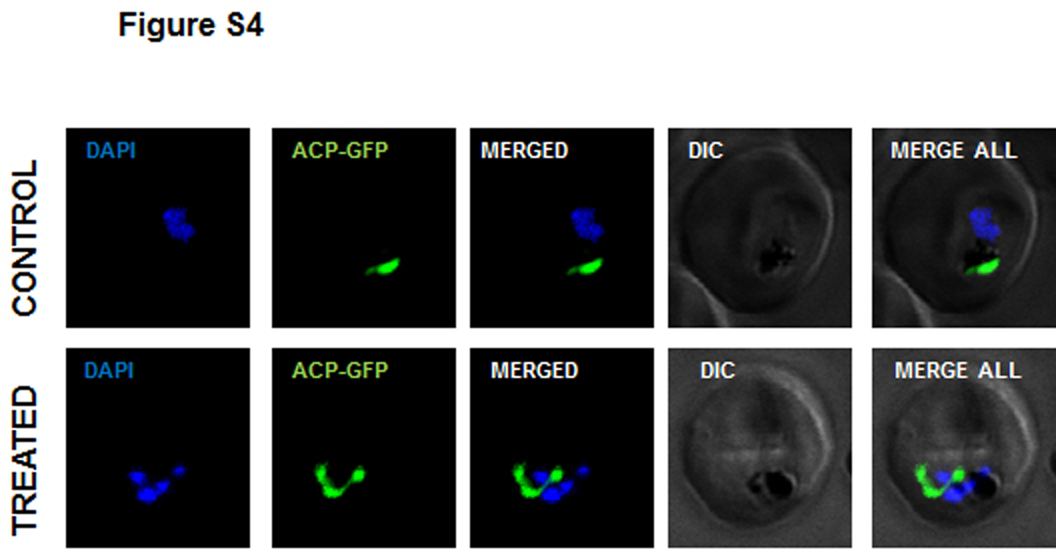

Supplement: Supplementary Figure 4 [file cddis2015142x4.tif]

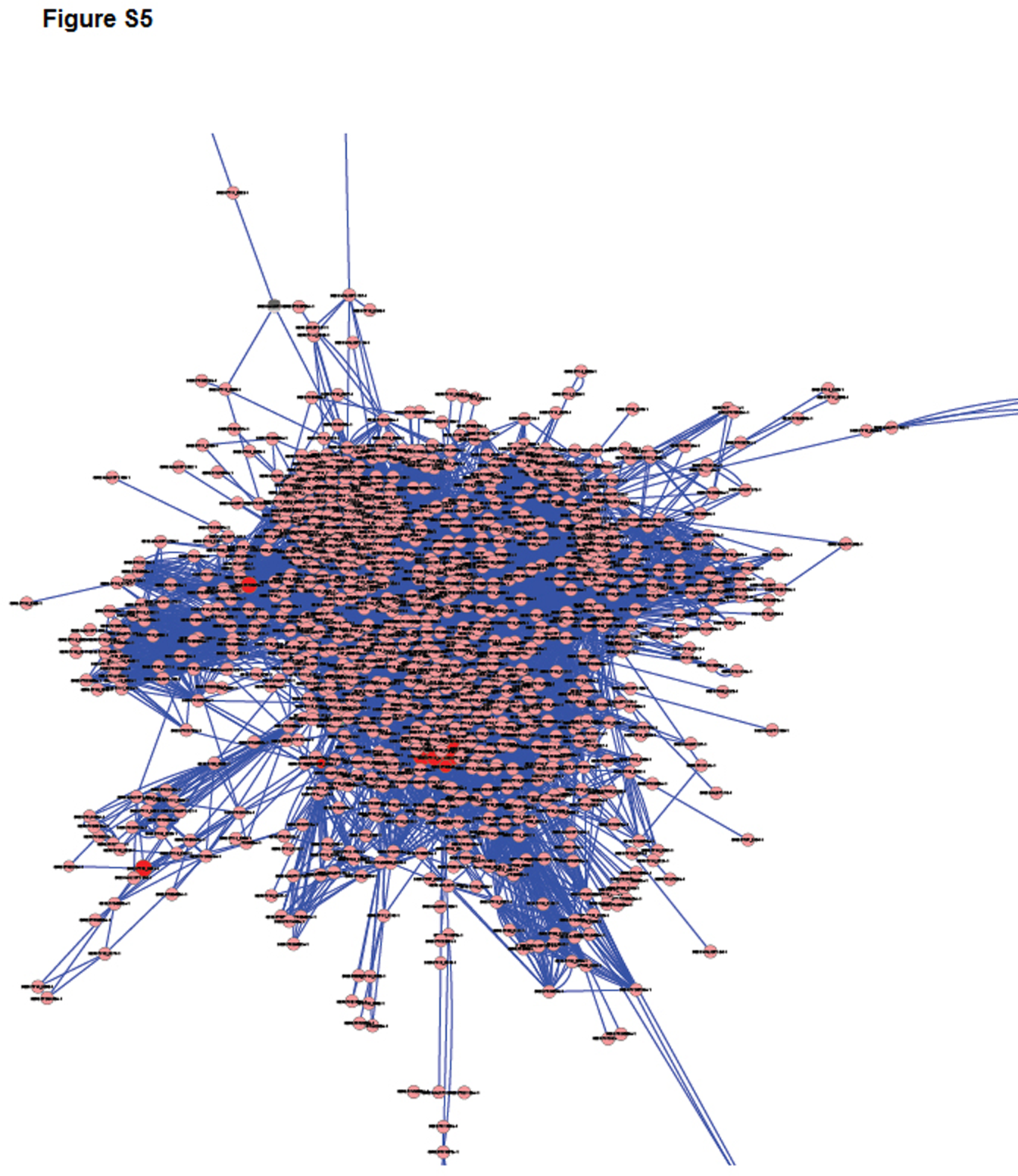

Supplement: Supplementary Figure 5 [file cddis2015142x5.tif]

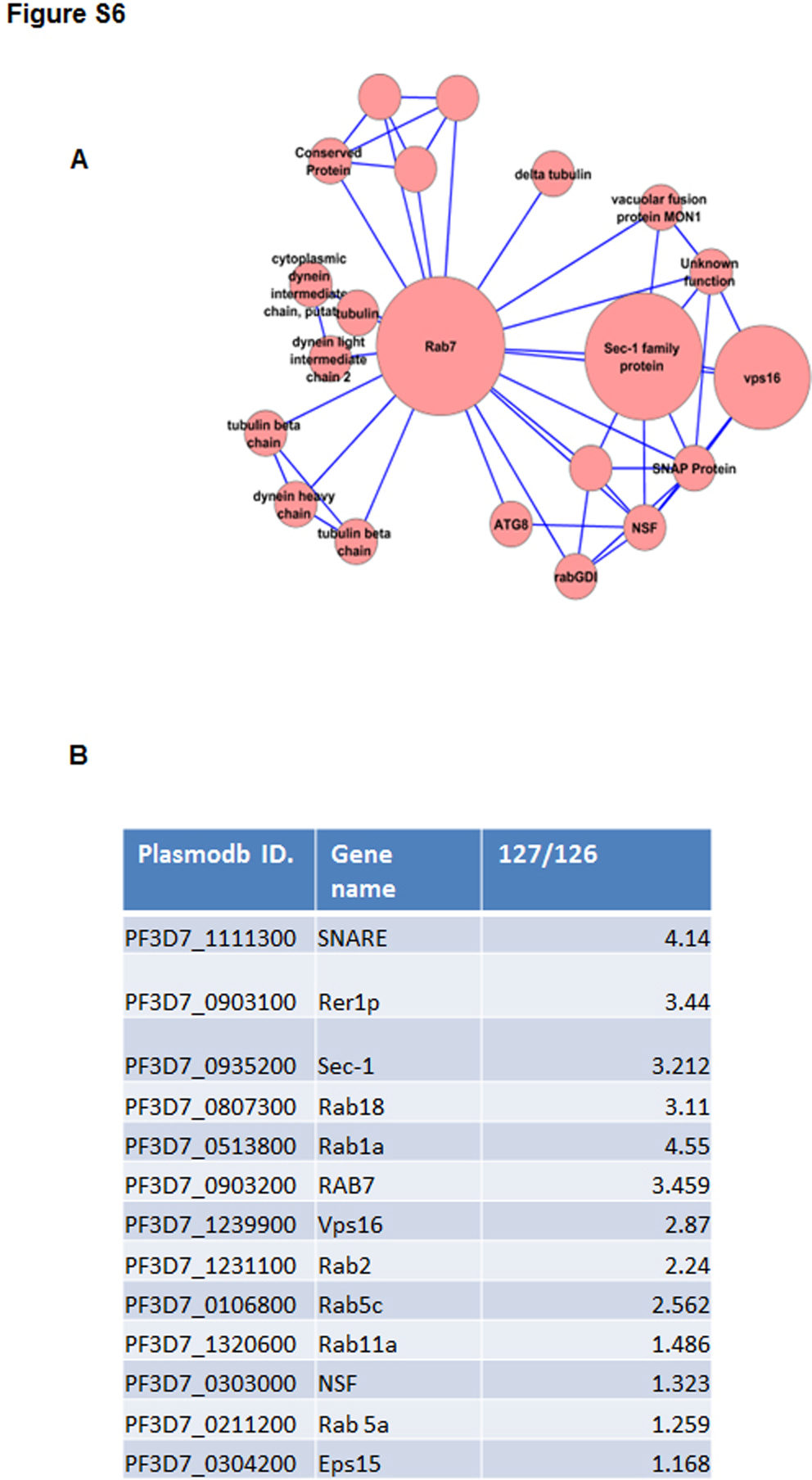

Supplement: Supplementary Figure 6 [file cddis2015142x6.tif]

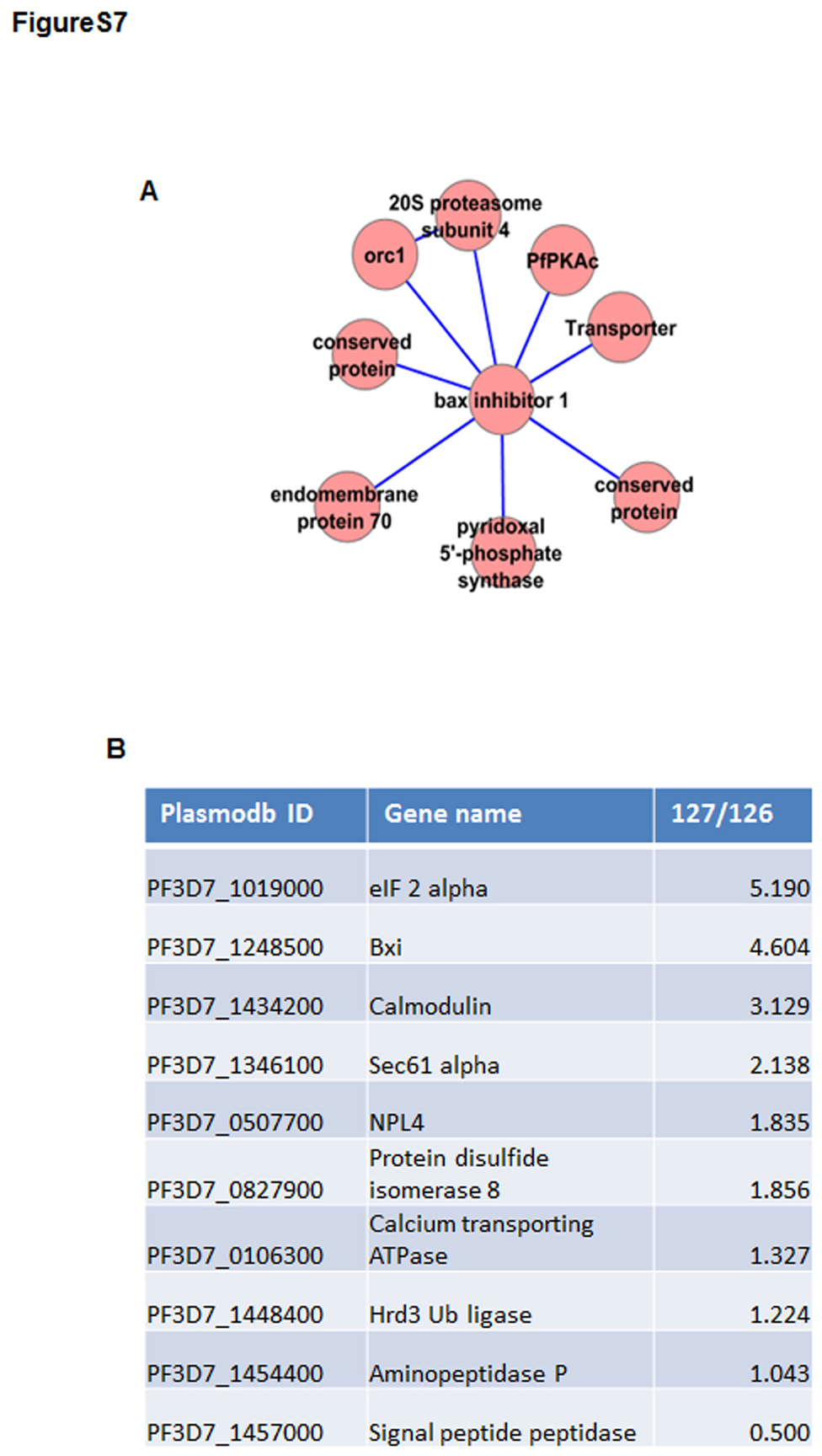

Supplement: Supplementary Figure 7 [file cddis2015142x7.tif]
