# Supplementary material for: Disruption of cellular homeostasis induces organelle stress and triggers apoptosis like cell-death pathways in malaria parasite
Source: Cell Death Dis. 2015 Jul 2;6(7):e1803–. doi: 10.1038/cddis.2015.142 (PMC4650714; doi:10.1038/cddis.2015.142)
Supplement: Supplementary Figure Legends [file cddis2015142x8.docx]

**Supplementary Figures**

**Figure S1. Effect of MG132 on growth of *P. falciparum*.** Tightly synchronised ring stage cultures (~14 hpi) were treated with different concentrations of MG132 or solvent alone. **A:** Line graph showing percentage parasite growth as estimated by formation of new ring stage parasites compared to control. **B:** Parasite composition at different time points (20-52 hpi) after MG132 treatment; the percentage of parasites in each morphological sub-group is indicated. **C:** Parasite morphology at different time points (28-54 hpi) after MG132 treatment (50 nm). **D:** Reversal of growth inhibition by removal of MG132 at different time points after treatment; the parasite samples were washed at different time points after MG132 treatment and then allowed to grow for 40 hrs. Percentage of new ring stage parasites were counted, and percentage growth rescue was estimated as compared to unwashed culture **p*<0.05, ***p*<0.01 and *** *p*<0.001.

**Figure S2. Release of calcium into parasite cytosol MG132 from ER after MG132 treatment. A.** Change in Mag-Fluo-4 fluorescence over time after MG132 treatment. Images captured prior to drug addition (0 seconds) and at the end of the experiment (3000 seconds) are shown on the right. The graph is an average of different parasites (n=20) from three independent experiments. **B.** Food-vacuole calcium concentration is maintained during MG132 treatment as shown by Fluo-4 staining. The bar graph on the right shows the summary of Mag-Fluo-4 and Fluo-4 staining, representing the average data from different parasites (*n*=20) from three independent experiments. **C.** Calcium flux in the cytoplasm followed by Fura Red staining after MG132 treatment. Free calcium is detected by excitation at 405 nm, and the bound form at 488 nm. Being a calcium quenching ionophore, fluorescence at 405 nm decreases upon Ca binding. However the ratio F (405/488) increases with rising cytoplasmic calcium **p*<0.05, ***p*<0.01 and *** *p*<0.001.

**Figure S3**. (A) Activation of CaspACE FITC-VAD-FMK binding cystein proteases in *P. falciparum* parasite. Flow cytometry dot blots for parasite population showing CaspACE tagged parasites in the cultures at different time points (2, 4, 6, and 8h) after treatment with MG132 or solvent alone. Percentage of CaspACE tagged parasite is also shown. (B) Expression PfMCA1 in HUH-7 cells expressing PfTSN-C1 and PfTSNC2 probed with anti-FLAG antibody.

**Figure S4**. Fluorescent images of transgenic *P. falciparum* D10-ACP parasites (expressing GFP fused to apicoplast resident acyl-carrier protein) treated with MG132 or solvent alone (control) showing no change in apicoplast morphology at 4 hrs; the parasite nuclei were stained with DAPI (blue).

**Figure S5**. Overview of the Protein-Protein interaction (PPi) network of all the detected proteins in quantitative global proteomic analysis of parasites after MG132 treatment (4h). Different functional clusters identified by MCode pluggin are indicated.

**Figure S6**. Proteins within functional cluster for “vesicular transport” proteins. (A) STRING based Protein-Protein interaction (PPi) network showing cluster of proteins involved in vesicular trafficking. The strength of each interaction is indicated by thickness of the connecting line. Size of nodes corresponds to the change in the levels after MG132 exposure. (B) Table showing change in levels (127/126 ratio) of each of the vesicular transport related proteins of this PPi network.

**Figure S7**: Proteins-Protein interaction network for “ER related proteins”. (A) String based Protein-Protein interaction network showing cluster of proteins involved in ER maintenance and function. The strength of each interaction is indicated by thickness of the connecting line. Size of nodes corresponds to the change in the levels after exposure. (B) Table showing change in levels (127/126 ratio) of each of ER related proteins in this PPi network.
